# Oversensing caused by trapped air in the header of subcutaneous cardioverter‐defibrillator generator

**DOI:** 10.1002/joa3.12912

**Published:** 2023-08-22

**Authors:** Takuya Okada, Junji Morita, Takayuki Kitai, Tsutomu Fujita, Yusuke Kondo

**Affiliations:** ^1^ Department of Clinical Engineering, Sapporo Heart Center Sapporo Cardiovascular Clinic Sapporo Japan; ^2^ Department of Cardiovascular Medicine, Sapporo Heart Center Sapporo Cardiovascular Clinic Sapporo Japan; ^3^ Department of Cardiovascular Medicine Chiba University Graduate School of Medicine Chiba Japan

**Keywords:** header air, inappropriate shock, loose pin, oversensing, subcutaneous implantable cardioverter‐defibrillator

## Abstract

In this study, we report two cases with oversensing due to air accumulation in the subcutaneous implantable cardioverter‐defibrillator (S‐ICD) device generator header. If trapped air in the header of the device is suspected, the re‐connection procedure should be considered or the primary vector must be used, which might prevent oversensing.

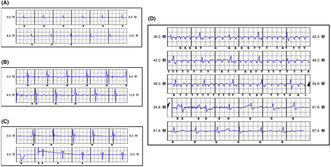

Oversensing caused by trapped air in the subcutaneous implantable cardioverter‐defibrillator (S‐ICD) generator header is rarely reported. We report two cases in which air trapped in the generator header resulted in oversensing.

A 52‐year‐old man with a S‐ICD was admitted to our hospital for an exchange of the generator. During the procedure, a lead was inserted, and normal function of the device was immediately achieved. A loose pin was not observed, but the impedance was within normal value when the tag test was performed. However, before closure of the incision, noise was observed on the secondary and alternate vectors of the electrogram tracing with a normal tracing on the primary vector (Figure 1A–C). The terminal pin was not recognized to be loose in correct position when visually confirming the header. The noise was eliminated when a torque wrench was inserted into the seal plug as we suspected air entrapment.

**FIGURE 1:**
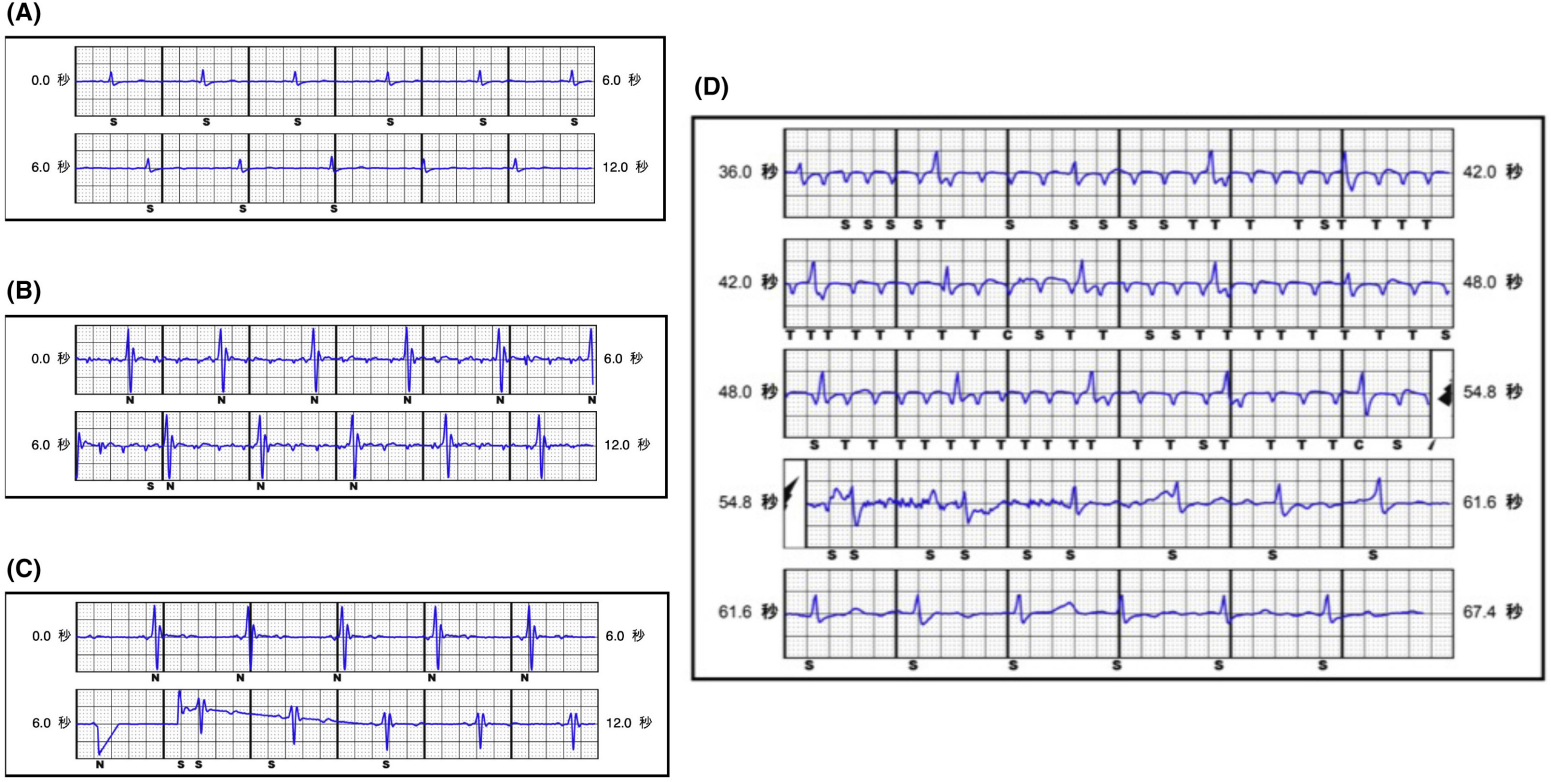
Sensing noise observed on the secondary and alternate vectors. (A) Primary vector, (B) Secondary vector, and (C) Alternate vector. (D) Inappropriate shock delivery owing to noise oversensing. C, charge start; N, noise; S, sense; T, tachy‐detection.

A 55‐year‐old man was referred to our center for implantation of an S‐ICD device for secondary prevention of VF. During the implantation, the automatic programming algorithm selected the secondary vector for sensing and a defibrillation test was successful. Normal function of the device was achieved immediately after the implantation on postoperative day one. However, 5 weeks after implantation, the device delivered a shock. Assessment of the device showed normal function with satisfactory sensing. The stored electrogram confirmed that an inappropriate shock was delivered owing to oversensing (Figure 1D). Specifically, flutter‐like waves and small amplitude drifting above the baseline were observed, which resulted in inappropriate shock delivery. As no T‐wave oversensing was detected, we hypothesized that the drifting waves were caused by a bad connection of the lead as a result of the pressure generated by the trapped air in the header of the device. However, chest radiographs and fluoroscopy did not reveal any trapped air or any evidence that the pin was not properly placed when the connection was completed. There was no dislocation of the lead or device. Moreover, the oversensing was not reproducible with any postural change or left arm isometric exercises. After the device was reprogrammed to the primary vector to avoid oversensing, remote monitoring over the next 2 years revealed no further abnormal discharge.

Air entrapment in the subcutaneous tissue surrounding the tip, ring, or the device itself in the pocket is also a well‐known reason for inappropriate shock early after S‐ICD implantation.^1^ However, oversensing due to air entrapment in the header of the S‐ICD has seldom been recognized as a cause of inappropriate S‐ICD discharge. A previous study identified air leakage through a seal plug damaged at the time of lead insertion and reported the leakage as a cause of oversensing of the S‐ICD system.^2^ In case 1, without re‐insertion of the lead into the header, the noise disappeared as the torque wrench was inserted into the seal plug. We speculated that the oversensing maybe caused by air entrapment inside the header port. Contrastingly, in case 2, oversensing occurred 5 weeks after the S‐ICD implantation, and was caused by fluttering waves drifting above the baseline. Such fluttering waves have previously been described,^3^ including irregularity in the morphology and amplitude of these waves with a varying degree of superimposition of the QRS complexes of the flutter‐like waves on the R wave. In case 2, there was no evidence of subcutaneous air entrapment or a loose pin when we visually examined the lead connection, but there might be loose contact of the electrode inside to lead to noises. From the two cases we presented, air entrapment in the header was the possible reason, which may have originated from the techniques of the lead connection. The steps of the lead connection process performed in our facility previously were different from the instructions recommended by the manufacturer, which are described below. The torque wrench was not inserted into the seal plug first, but was kept in position throughout the lead connection process. On the contrary, the lead was inserted into the port before the torque wrench insertion, and the torque wrench was only inserted when the set screw was turned to fix the lead. Therefore, we speculated that the air entrapped in the lead port may be compressed to create higher pressure, which eventually pushed the lead from the ideal connection position gradually. The loose contact of the terminal pin may explain why the noise was observed on the secondary and alternate vectors, but not on the primary vector. The sensing vector of S‐ICD is shown (Figure 2A). When air stays in the distal sensing electrode (Figure 2B①), noise is added to the alternate and secondary, but this time the same thing happens when air remains in the generator header. This is because the terminal pin (Figure 2B⑨), which is the connection between the main unit and the lead, is part of the distal sensing electrode. Therefore, when air is trapped in the header or the seal plug, noise is recognized in the same way as when air stays in the distal sensing electrode. Additionally, we conducted a bench test using a water tank. After inserting the lead into the device header, a torque wrench was inserted into the seal plug in the water tank to fix the terminal pin. When the torque wrench was inserted again to check the residual air, an air bubble was confirmed to be released from the seal plug (Figure 3A). Then, pushing the lead further released a small amount of air (Figure 3B). From this, it is considered that a small amount of air will remain in the header if the lead is inserted in this procedure and the lead is not pushed in after inserting the torque wrench.

**FIGURE 2:**
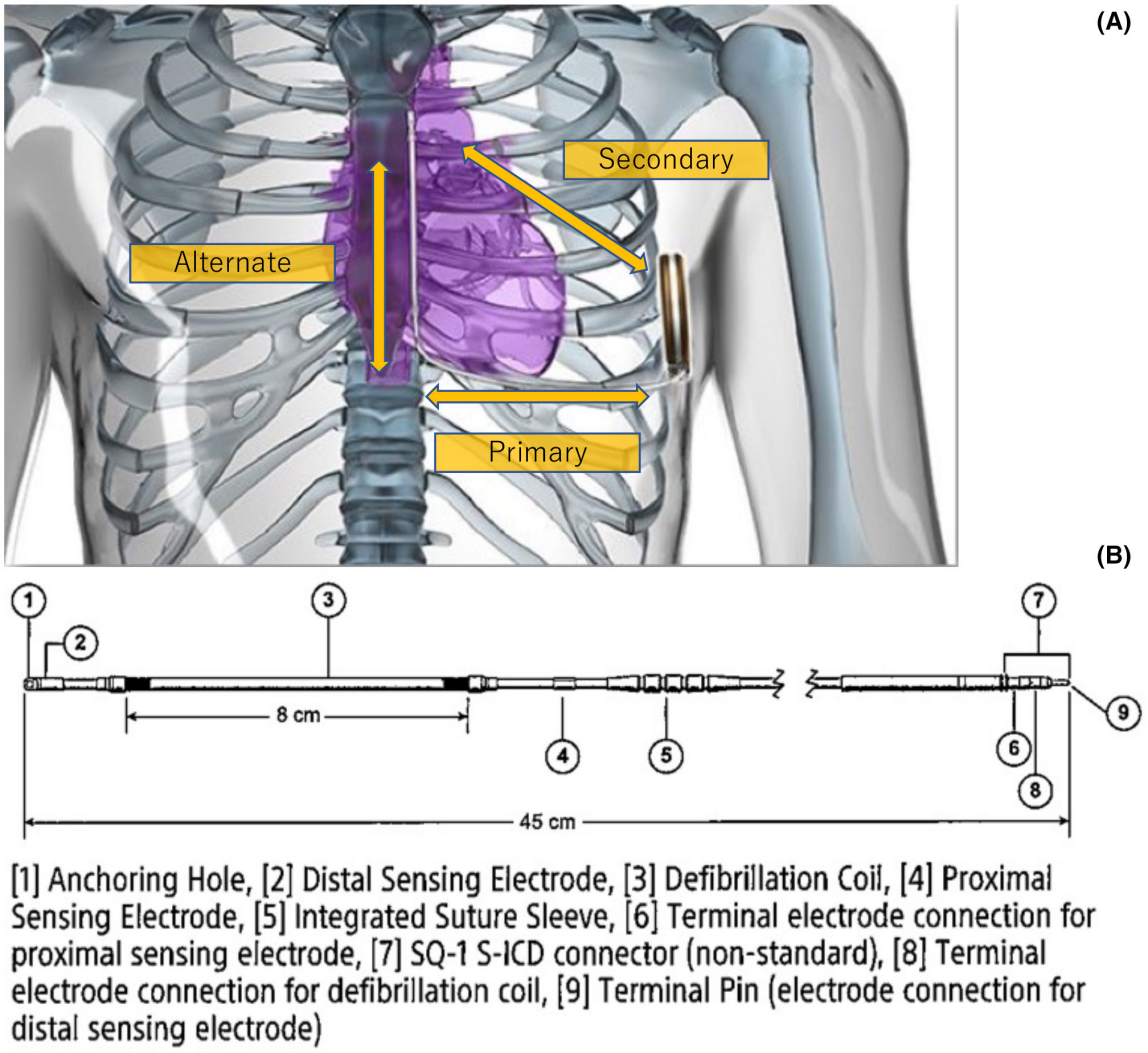
(A) Vector. (B) ① Anchoring Hole ② Distal Sensing Electrode ③ Defibrillation Coil ④ Proximal Sensing Electrode ⑤ Integrated Suture Sleeve ⑥ Terminal electrode connection for proximal sensing electrode ⑦ SQ‐1 S‐ICD connector (nonstandard) ⑧ Terminal electrode connection for defibrillation coil ⑨ Terminal Pin (electrode connection for distal sensing electrode).

**FIGURE 3:**
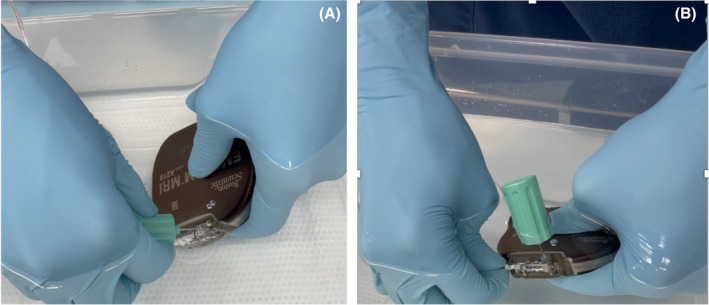
(A) Bench testing of the association between trapped air in the header of the device and the lead insertion maneuver performed in the water tank. (B) Released a small amount of air.

Trapped air in the generator header is more difficult to determine radiographically and visually than air in other parts of S‐ICD. Differentiation from atrial tachycardia and atrial flutter was performed as follows. In the first case, we changed the vector and determined that there was no noise in primary vector, so we confirmed that the noise was caused by air in the header. In the latter case, a detailed reading of the waveform revealed that the cycle length was repetitive shortening and lengthening, which is atypical for AFL, and we determined the waveform was air noise. The two cases demonstrate the possibility of header air of S‐ICD, leading to oversensing. If trapped air in the header is suspected after implantation, changing the vector to primary could prevent oversensing.

## CONFLICT OF INTEREST STATEMENT

Dr. Kondo received lecture fees from Daiichi‐Sankyo, Bayer, Abbott Medical Japan, Biotronik Japan, Boston Scientific, and Japan Lifeline, and research funds from Daiichi‐Sankyo. The other authors have no conflict of interest to declare.

## ETHICS APPROVAL STATEMENT

This study was conducted according to the principles of the Declaration of Helsinki. The study was approved by the Institutional Review Board.

## PATIENT CONSENT STATEMENT

The patients provided written informed consent.
